# Impact of Inhibition of Glutamine and Alanine Transport on Cerebellar Glial and Neuronal Metabolism

**DOI:** 10.3390/biom12091189

**Published:** 2022-08-27

**Authors:** Abhijit Das, Gregory Gauthier-Coles, Stefan Bröer, Caroline D. Rae

**Affiliations:** 1 Neuroscience Research Australia, Barker St, Randwick, NSW 2031, Australia; 2 School of Medical Sciences, The University of New South Wales, Sydney, NSW 2052, Australia; 3 Department of Pharmacy, Noakhali Science and Technology University, Noakhali 3814, Bangladesh; 4 Division of Biomedical Science and Biology, Australian National University, Canberra, ACT 2600, Australia; 5 School of Psychology, The University of New South Wales, Sydney, NSW 2052, Australia

**Keywords:** glutamine, alanine, amino acid transporters

## Abstract

The cerebellum, or “little brain”, is often overlooked in studies of brain metabolism in favour of the cortex. Despite this, anomalies in cerebellar amino acid homeostasis in a range of disorders have been reported. Amino acid homeostasis is central to metabolism, providing recycling of carbon backbones and ammonia between cell types. Here, we examined the role of cerebellar amino acid transporters in the cycling of glutamine and alanine in guinea pig cerebellar slices by inhibiting amino acid transporters and examining the resultant metabolism of [1-^13^C]d-glucose and [1,2-^13^C]acetate by NMR spectroscopy and LCMS. While the lack of specific inhibitors of each transporter makes interpretation difficult, by viewing results from experiments with multiple inhibitors we can draw inferences about the major cell types and transporters involved. In cerebellum, glutamine and alanine transfer is dominated by system A, blockade of which has maximum effect on metabolism, with contributions from System N. Inhibition of neural system A isoform SNAT1 by MeAIB resulted in greatly decreased metabolite pools and reduced net fluxes but showed little effect on fluxes from [1,2-^13^C]acetate unlike inhibition of SNAT3 and other glutamine transporters by histidine where net fluxes from [1,2-^13^C]acetate are reduced by ~50%. We interpret the data as further evidence of not one but several glutamate/glutamine exchange pools. The impact of amino acid transport inhibition demonstrates that the cerebellum has tightly coupled cells and that glutamate/glutamine, as well as alanine cycling, play a major role in that part of the brain.

## 1. Introduction

Glutamine homeostasis in the brain is of key importance for the maintenance of both excitatory and inhibitory functions. Glutamine is a major amino acid source for the synthesis of both glutamate and GABA. It serves as a neutralised precursor of both neurotransmitters; glutamate released from neurons can be converted to glutamine in glial cells and returned to the neuron in that form [1,2,3]. Similarly, much of the GABA synthesised in GABAergic neurons is derived from astrocytic glutamine [4,5,6]. As classically written, the glutamate/glutamine cycle is not stoichiometric [7] but interacts with other pathways including transaminase catalysed reactions. Return of ammonia from neuron to astrocyte, for example, is likely accomplished through these reactions, carried by cycling amino acids such as alanine [8,9,10,11].

Release and uptake of glutamine are accomplished by an array of amino acid transporters (Table 1). Recent cloning and characterization of Gln transporters along with in vitro cell culture studies have demonstrated that Gln transport in astrocytes and neurons mainly involves five amino acid transport systems, namely A, ASC, N, B^0^, and L [12,13,14,15,16,17,18]. Gln efflux from astrocytes into the extracellular fluid is mediated by system N-like transporters [16] while system A-, B^0^-, and L-like transporters may contribute to neuronal uptake [13,19,20,21,22,23]. Several gene products may contribute to each of these physiologically characterised amino acid transport systems [24].

Uptake and exchange of alanine in the brain are similarly undertaken by an array of transporters with submillimolar affinity for alanine including systems A, L and B^0^ (Table 1). Alanine is produced from pyruvate via alanine aminotransferase (ALT, L-alanine:2-oxoglutarate aminotransferase E.C.2.6.1.2) which has mitochondrial and cytosolic forms [25] and which has been shown to incorporate NH_4_ from glutamate provided as ^15^N glutamine [26]. 

SNAT3 or SN1 (SLC38A3), an isoform of amino acid transport system N [27,28], is exclusively expressed in astrocytes [20,29] and regulates Gln movement (both influx and efflux) from astrocytes. ASCT2 (SLC1A5), belonging to the ASC transport system, is highly expressed in reactive and cultured astrocytes but is only found in small amounts in the intact brain [15,19,30]. It mediates the exchange of small and polar neutral amino acids [15,31,32] but ASCT2 (SLC1A5) is not found to be expressed in the cerebellum (Table 2). 

SNAT1 (also known as GlnT or ATA1; SLC38A1) and SNAT2 (also known as SAT2 or ATA2; SLC38A2) are two molecular correlates of transport system A which are localized in neurons [19,33,34,35] and predominantly regulate neuronal Gln uptake [20,36,37]. SNAT2, the preferred substrate of which is alanine [35], is also found to be expressed in oligodendrocytes [38]. Two transporters from the system B^0^ transporter family, B^0^AT2 (also called SBAT1; SLC6A15) and NTT4 (also called XT1/B^0^AT3; SLC6A17), are exclusively expressed in glutamatergic and GABAergic neurons [18,21,22,39,40] and transport Gln along with other neutral amino acids into neurons [21,22]. Apart from these, several transporters in the system L transport family such as LAT1 (SLC7A5), LAT2 (SLC7A8) and LAT4 (SLC43A2) are responsible for the exchange of large neutral and aromatic amino acids across the plasma membrane [17,41,42] and they are expressed both in astrocytes and neurons [2,43,44,45,46].

While the five amino acid transport systems named above, A, ASC, N, B^0^, and L, are generally regarded as the major carriers for glutamine uptake in the mammalian brain, not all of the transporters belonging to these systems are expressed in the cerebellum. Our current knowledge concerning glutamine transport in the cerebellum, based on previous studies [2,18,19,21,58,68] showed that there are eight transporters which are expressed in the cerebellum and involved in the uptake and release of glutamine from neurons and astrocytes (Table 1). The pharmacology of glutamine transporters is underdeveloped [73]; however, histidine is a substrate of all glutamine transporters and can thus be used to compete with the uptake of glutamine through all routes. Another non-specific glutamine analogue is γ-glutamyl-p-nitroanilide (GPNA), while cycloleucine serves as an analogue of branched-chain amino acids. More specific inhibitors are available for system A (MeAIB) and system L (BCH). The glutamine analogue AABA [56] is an inhibitor of system L, B^0^ and SNAT2.

Much of our information about glutamine cycling in the brain has been gleaned from the cortex but there is evidence that the energy budget and the distribution of excitatory and inhibitory neurons in the cerebellum differs significantly from that in the cortex. According to Howarth et al., (2012), the total energy use for the cerebral cortex is 27.2–40.7 µmol ATP/g/min compared to that of 17.1–25.6 µmol ATP/g/min for the cerebellar cortex. In terms of energy expenditure, the cerebral cortex utilizes 21% of its signalling energy on action potentials, and 50% on postsynaptic glutamate receptors, whereas 17% of cerebellar signalling energy is used on the maintenance of action potentials and 22% on postsynaptic receptors [74]. Work with cultured cerebellar astrocytes has shown that glutamate/glutamine cycling is tightly coupled in these preparations with fast removal of glutamate from the synapse [75] while other work has shown other cerebellar synapses which maintain extracellular physiologically relevant levels of glutamate to regulate both tonic and phasic activity [76]. A recent study by Ferreira et al., (2021), using ex vivo ^1^H-and ^13^C-NMR spectroscopy, demonstrated that the concentration of several metabolites (including glutamine) in rat cerebellum was distinctly different from that in the cerebrum [77,78]. Despite these differences in metabolite concentrations and energy expenditure in the cerebellum, no satisfactory understanding of neurochemical mechanisms underlying glutamine metabolism in this part of the brain has been achieved to date.

To investigate the roles played by the different amino acid transporters in glutamine and alanine exchange in the cerebellum, we employed a guinea pig brain cerebellar tissue slice preparation [79] incubated with [1-^13^C]d-glucose and [1,2-^13^C]acetate in the absence (control) and presence of competitive inhibitors of the different glutamine transporters, mimicking a successful approach previously employed in the cortex to investigate glutamine [30] and alanine [9] transport. Acetate is mostly metabolised in glial cells [80] although there is also evidence that the neuronal metabolism of acetate is not insignificant [81,82]. To stimulate glutamine cycling, these incubations were done under depolarising (40 mm K^+^) conditions.

## 2. Materials and Methods

### 2.1. Materials

A total of 24 guinea pigs (Dunkin-Hartley), both male and female (obtained from Pipers Farm, NSW, and Australia and Flinders University, Australia respectively) and weighing about 400–800 g were used in the experiments. They were fed ad libitum on standard Guinea pig/rabbit pellets, with fresh carrots and hay roughage, and maintained a 12 h light/dark cycle. All experiments were carried out according to the guidelines of the National Health and Medical Research Council of Australia and were approved by the institutional (UNSW) Animal Care Ethics Committee (ethical approval number: 20/105B, Approval date: 1 July 2020). The study, an exploratory investigation, was not pre-registered. 

[1-^13^C]d-glucose, [1,2-^13^C]acetate and sodium [^13^C]formate were purchased from Cambridge Isotope Laboratories Inc (Andover, MA, USA). Histidine, MeAIB (2-(methylamino)isobutyric acid), cycloleucine (1-aminocyclopentanecarboxylic acid), BCH (2-aminobicyclo [2.2.1]heptane-2-carboxylic acid) and GPNA (L-γ-glutamyl-p-nitroanilide) were purchased from Sigma-Aldrich (St Louis, MO, USA). AABA (2-amino-4-bis(aryloxybenzyl)aminobutanoic acid; compound 12) was supplied by Professor Stefan Bröer (Research School of Biology, Australian National University) [56]. All other reagents were of Analytical Reagent grade.

### 2.2. Preparation of Brain Cerebellar Tissue Slices

Cerebellar tissue slices were prepared from guinea pigs following the previously described protocol [79]. Guinea pigs were sacrificed in the mid-morning by cervical dislocation and the brain was immediately removed. Then, the cerebellum was dissected and chopped into 350 µm slices in the parsagittal plane using a McIlwain tissue chopper (The Mickle Laboratory Engineering Company, Gomshall, Surrey, UK) and the resulting slices were instantly washed three times in a modified Krebs-Henseleit buffer (124 mm NaCl, 5 mm KCl, 1.2 mm KH_2_PO_4_, 1.2 mm CaCl_2_, 1.2 mm MgSO_4_ and 26 mm NaHCO_3_) [83] containing 10 mm d-glucose. Cerebellar tissue slices were then resuspended for 1 h in fresh buffer containing 10 mm d-glucose and gassed with 95% O_2_/5% CO_2_ in a shaking water bath, maintained at 37 °C, to allow metabolic recovery [84]. Slices were then combined and washed three times in glucose-free modified Krebs-Henseleit buffer and resuspended in separate experimental flasks containing fresh buffer along with the different substrates of choice [79].

### 2.3. Inhibition of Glutamine Transport

To evaluate the relative effects of inhibition of different glutamine transporters on metabolism, the following experiments were performed using recovered Guinea pig cerebellar tissue slices:

Incubation of 5.0 mm [1-^13^C]d-glucose and 0.5 mm [1,2-^13^C]acetate (control) with 10 mmol/L histidine (to block systems A, L and N) [30,60]. Cerebellar slices from four male guinea pigs were used in this experiment.

Incubation of 5.0 mm [1-^13^C]d-glucose and 0.5 mm [1,2-^13^C]acetate (control) with 10 mmol/L MeAIB (to block system A) [9,30]. Although MeAIB has been reported to block system L at 10 mm MeAIB there is only a 20% reduction in uptake of glutamine through this system indicating that the K_i_ for MeAIB is well in excess of 10 mm used here [85]. Cerebellar slices from four male guinea pigs were used in this experiment.

To test the effect of blocking systems A and L, chopped cerebral cortices were incubated with 5.0 mm [1-^13^C]d-glucose and 0.5 mm [1,2-^13^C]acetate (control) and with 100 µmol/L of the potent inhibitor AABA [56]. The chopped cerebella of four male guinea pigs were used for this experiment.

Cerebellar tissue slices were incubated with 5.0 mm [1-^13^C]d-glucose and 0.5 mm [1,2-^13^C]acetate (control) and with 10 mmol/L GPNA to block systems A, ASC and L [55,64,65]. Here, we incubated the chopped cerebella of four female guinea pigs with the substrates.

In order to block system L, recovered brain slices were incubated with 5.0 mm [1-^13^C]d-glucose and 0.5 mm [1,2-^13^C]acetate (control) and with 10 mmol/L cLeu [9]. The chopped cerebella of four female guinea pigs were used for this experiment.

In order to block system L and B^0^, recovered brain slices were incubated with 5.0 mm [1-^13^C]d-glucose and 0.5 mm [1,2-^13^C]acetate (control) and with 10 mmol/L BCH [9]. Chopped cerebellar slices from four female guinea pigs were used for this experiment.

All experiments were carried out under conditions of functional activation, i.e., depolarization with 40 mm KCl [83] in order to stimulate glutamate/glutamine cycling as described previously [9,30]. Slices were incubated for 30 min under these conditions and the experiment stopped as described below.

### 2.4. Preparation of Samples

On completion of the incubation period, slices were removed from the incubation buffer by rapid filtration and were frozen in liquid nitrogen. Pulverised frozen tissue was double extracted using chloroform/methanol [86]. The aqueous phase was lyophilised and reconstituted in ^2^H_2_O containing 2 mm sodium [^13^C]formate as an internal intensity reference, and 2 mm EDTA as a chelating agent to remove paramagnetic species, while the resulting pellet was dried and retained for protein estimation as above [87].

### 2.5. NMR Analysis

^1^H, [^13^C]-decoupled ^1^H and [^1^H]-decoupled ^13^C NMR spectra were acquired from the guinea pig cerebellar slice extracts using a Bruker AVANCE III HD 600 NMR spectrometer equipped with a TCI cryoprobe and refrigerated sample changer, as described previously [81]. Peak areas were compared to the area of the [^13^C]-formate peak in the case of the ^13^C spectra and adjusted for saturation and nuclear Overhauser effect by reference to a standardised fully relaxed (TR = 90 s) ^13^C spectrum acquired with ^1^H decoupling only during the acquisition time. Peaks in the fully relaxed (TR = 30s) ^1^H spectrum were referenced to the area of the ^13^C satellites from the known concentration of [^13^C]-formate. Values were expressed as μmol of metabolite per 100 mg protein where protein concentration was measured in the pellet obtained after extraction using the method of Lowry [88] as described previously [89].

### 2.6. Measurement of Metabolic Pool Sizes by LCMS

Metabolites from guinea pig cerebellar homogenates were analysed using two LCMS methods. Common across both methods was the sample preparation. Briefly, this consisted of transferring 40 μL of lyophilised cortex resuspended in water to microcentrifuge tubes containing 160 μL of methanol. Two hundred microlitres of chloroform was added and the tubes were vortexed for five minutes, cleared by centrifugation, after which 60 μL of the aqueous phase was transferred to one tube for analysis by HILIC separation and to another tube for derivatisation and analysis by reverse phase chromatography.

For the relative quantification of glutamine, samples were desolvated in a vacuum concentrator for three hours and resuspended in 10 mm ammonium acetate: acetonitrile (1:9 + 0.15% formic acid). Internal standards (MSK-A2-1.2 and CLM-1822-H-0.1; Cambridge Isotope Laboratories) were added at a final concentration of 10 μM. Amino acids were separated using a SeQuant ZIC cHILIC 3 μm 100Å 150 × 2.1 mm column (EMD Millipore, Burlington MA) fitted to an UltiMate 3000 RS UHPLC system (Dionex, Lane Cove West, Australia) coupled to an Orbitrap Fusion mass spectrometer (Thermo Fisher Scientific, Scoresby, Australia). The mobile phase consisted of solvent A (10 mm ammonium acetate + 0.15% formic acid) and solvent B (acetonitrile + 0.15% formic acid). The flow rate was set to 0.4 mL/min and the total run time was 21 min. Solvent A was set to 10% from 0 min to 6 min, increased linearly to 15% at 6.1 min, followed by a further increase to 26% at 10 min, and again to 36% at 12 min and finally to 64% at 12.1 min. Solvent A remained at 64% until 17 min before it was returned to 10% at 17.1 min and maintained until the end of the run to re-equilibrate the column. The column oven temperature was fixed to 35 °C and 4 μL of the sample was injected for each run. Sheath gas, auxiliary gas and sweep gas were set to 40, 5 and 1, respectively, (all arbitrary units). Ion transfer tube temperature was set to 300 °C and vaporizer temperature to 400 °C. Analytes were ionized in positive mode using heated electrospray ionization and the ion spray voltage was set to 3400 V. RF lens were set to 50% and the mass filter excluded ions outside the *m/z* range of 50–200. Analysis was performed in full scan mode (R = 50,000).

All other metabolites were analysed using a derivatization method whereby samples were desolvated in a vacuum concentrator for three hours, resuspended in 100 μL of 1-butanol with 3 N HCl, vortexed for five minutes and incubated at 65 °C for 30 min. Samples were once more desolvated and then resuspended in 10 mm ammonium acetate: acetonitrile (93:7 + 0.15% formic acid). Internal standards were prepared in the same way with the exception that 1-butan-d9-ol (615099; Sigma, Burlington, MA, USA) was used instead of 1-butanol and were added to the samples at a final concentration of 10μM. Analytes were separated using a Kinetex 1.7 μm C18 100Å 100 × 2.1 mm column fitted to an UltiMate 3000 RS UHPLC system (Dionex, Lane Cove West, Australia) coupled to an Orbitrap Fusion mass spectrometer (Thermo Fisher Scientific, Scoresby, Australia). The mobile phase solvents were the same as those described above. The flow rate was set 0.3 mL/min and the total run time was 22 min. Solvent A was set to 93% from 0 min to 4 min, linearly decreased to 20% at 12 min where it was held until 17 min, followed by an increase back to 93% at 17.1 min where it remained until the end of the run. All other parameters were the same as those described above, with the following exceptions: positive voltage was set to 3500 V; sheath gas flow to 50; auxiliary gas flow to 10; ion transfer tube to 325 °C; vaporizer temperature to 350 °C; and the mass filter set to 50–400 *m*/*z*.

Analyte detection and quantification were achieved using Xcalibur (Thermo Fisher Scientific, Scoresby, Australia) by comparing the area under the peak of each analyte and their respective internal standard. Blanks were injected intermittently throughout each sequence and external standards at the beginning and end.

### 2.7. Visualization of Expression Data

Expression data of mouse adult cerebellum [72] were analyzed using the DropViz online portal (Dropviz.org). Expression levels were filtered by gene ID and region (cerebellum). TSNE plots were used without further modification. Numerical expression data were copied from the table view as normalised mean log expression in that cell type compared to the rest of the cerebellum (Table 2).

### 2.8. Statistical Analysis

Researchers were not blinded to the nature of the samples but were naïve to protein values when determining outcomes from NMR and LC-MS. Final values were calculated by predetermined algorithms as described previously in a spreadsheet upon input of raw data.

The slice experiment is designed as a population, contributed to by slices from the stated number of guinea pigs, with repeated sampling (N = 4). Historical data from our lab shows effect sizes (Cohens D) from these experiments with N = 4 varying from 1.6 to 4 depending on the variable (metabolite) and the signal to noise of the resonance. No data were excluded.

All statistical analysis was done in SPSS (IBM Statistics, v22 Armonk, NY, USA). Variables from experiments with each inhibitor were compared with the values from that particular control experiment using the non-parametric Mann–Whitney U test. No adjustments were made for multiple comparisons due to the highly correlated nature of the metabolic data. Results were considered significant if *p* < 0.05. Data are presented as mean ± SD and are all N = 4.

## 3. Results

### 3.1. Inhibition of Glutamine Transport with 10 mm Histidine under Depolarizing Conditions

**Figure 1:**
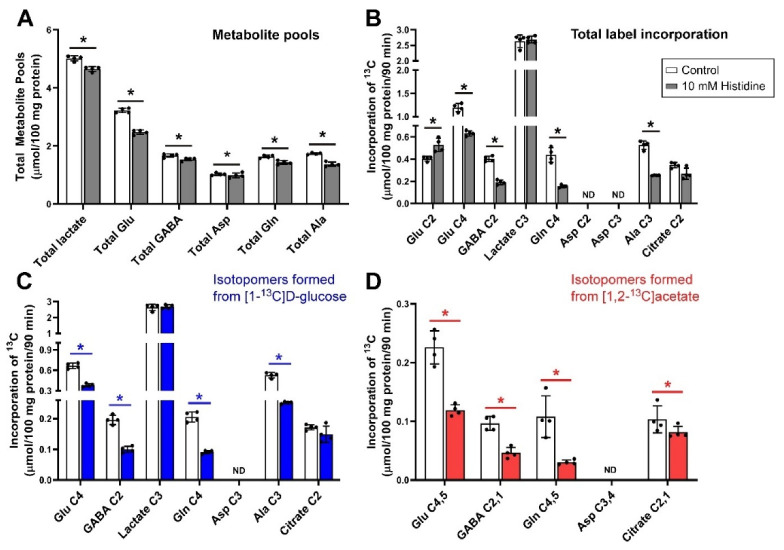
Metabolite pool sizes and net flux ^13^C label following incubation of cerebellar tissue slices with 5 mmol/L [1-^13^C]d-glucose and 0.5 mmol/L [1,2-^13^C]acetate (control) and 10 mmol/L histidine. (**A**), Total metabolite pools; (**B**), Total net fluxes of ^13^C; (**C**), Net fluxes derived from [1-^13^C]d-glucose; (**D**), Net fluxes derived from [1,2-^13^C]acetate. Bars show mean and error bars show standard deviations, N = 4. * *p* < 0.05 as shown with statistical significance (Mann–Whitney U test) between groups indicated by the line below the asterisk. ND = Not detected.

The effects of incubating brain cerebellar tissue slices with 10 mmol/L histidine in the presence of 5.0 mmol/L [1-^13^C]d-glucose and 0.5 mmol/L [1,2-^13^C]acetate under depolarizing conditions (40 mmol/L K^+^) are shown in Figure 1. Histidine significantly reduced total metabolite pool sizes of lactate, glutamate, GABA, aspartate, glutamine and alanine (Figure 1A). Net flux of ^13^C (i.e., label incorporated from either glucose or acetate substrates) into Glu C2 was significantly increased compared to control, while total labelling of Glu C4, GABA C2, Gln C4, and Ala C3 decreased (Figure 1B). There was no significant change in lactate C3 labelling indicating that glycolysis and the rates of pyruvate clearance were largely not impacted. Labelling of aspartate in this experiment with ^13^C was not detected above the signal to noise threshold.

### 3.2. Inhibition of Glutamine Transport with 10 mm MeAIB under Depolarizing Conditions

**Figure 2:**
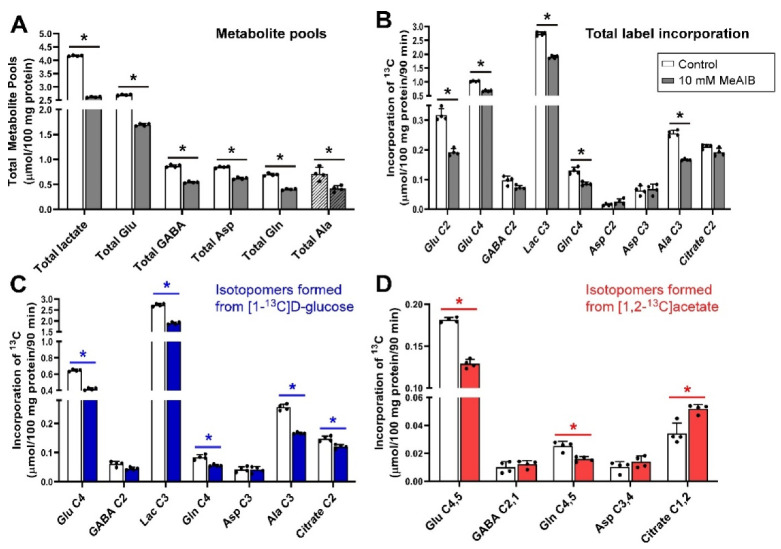
Distribution of ^13^C label following incubation of cerebellar tissue slices with 5 mmol/L [1-^13^C]d-glucose and 0.5 mmol/L [1,2-^13^C]acetate (control) and 10 mmol/L MeAIB. (**A**), Total metabolite pools; (**B**), Total net fluxes of ^13^C; (**C**), Net fluxes derived from [1-^13^C]d-glucose; (**D**), Net fluxes derived from [1,2-^13^C]acetate. Bars show mean and error bars show standard deviations, N = 4. * *p* < 0.05 as shown with statistical significance (Mann–Whitney U test) between groups indicated by the line below the asterisk. Hatched bars represent the pool sizes derived from LCMS.

The addition of 10 mmol/L MeAIB (which inhibits SNAT1, SNAT2 (Table 1)) to slices incubated with [1-^13^C]d-glucose and [1,2-^13^C]acetate significantly decreased the pool sizes of all measured metabolites (Figure 2). Incorporation of ^13^C label into Glu C2, Glu C4, lactate C3, Gln C4 and Ala C3 (all *p* = 0.029) was significantly reduced, indicating that glutamate/glutamine cycle activity was decreased, as was net flux through the Krebs cycle and glycolysis. In contrast to histidine, there was no significant effect on net flux into GABA C2, and citrate C2 (Figure 2B). Net flux of ^13^C derived from [1-^13^C]d-glucose reflected the changes in total net flux apart from reduced net flux into citrate C2 (*p* = 0.029) while Asp C3 was not significantly changed (Figure 2C).

In contrast to the decrease in net flux from [1-^13^C]d-glucose into citrate C2, the amount of ^13^C from [1,2-^13^C]acetate incorporated into citrate C2,1 was significantly increased by the presence of MeAIB while Glu C4,5 and Gln C4,5 showed significant reductions in label incorporation from [1,2-^13^C]acetate while there was no impact on label incorporation into GABA C2,1 (Figure 2D).

Taken together, these results indicate that blockade of Gln transport by MeAIB via system A transporters (i.e., SNAT1 and SNAT2 caused a reduction in neuronal Gln supply leading to widespread loss of metabolite pools.

### 3.3. Inhibition of Glutamine Transport with AABA 100 µm under Depolarizing Conditions

**Figure 3:**
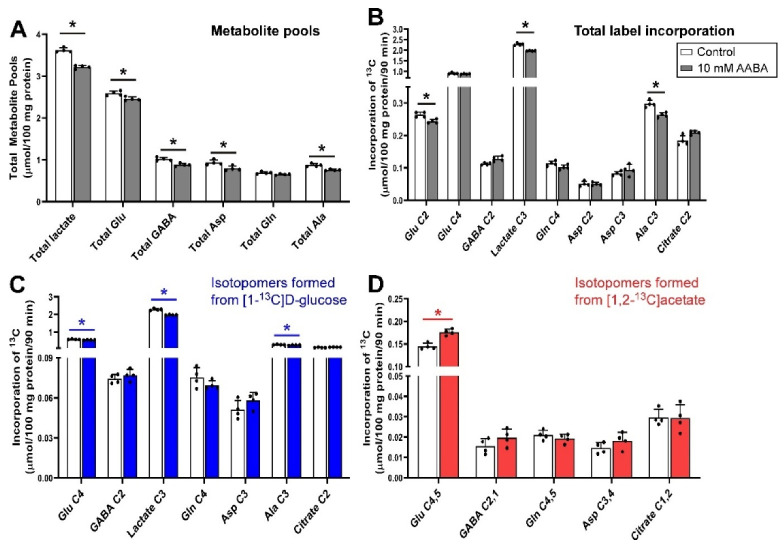
Distribution of ^13^C label following incubation of cerebellar tissue slices with 5 mmol/L [1-^13^C]d-glucose and 0.5 mmol/L [1,2-^13^C]acetate (control) and 100 μmol/L AABA. (**A**), Total metabolite pools; (**B**), Total net fluxes of ^13^C; (**C**), Net fluxes derived from [1-^13^C]d-glucose; (**D**), Net fluxes derived from [1,2-^13^C]acetate. Bars show mean and error bars show standard deviations, N = 4. * *p* < 0.05 as shown with statistical significance (Mann–Whitney U test) between groups indicated by the line below the asterisk.

The metabolic outcomes of incubating guinea pig cerebellar brain tissue slices with 100 μmol/L AABA in the presence of 5 mmol/L [1-^13^C]d-glucose and 0.5 mmol/L [1,2-^13^C]acetate are shown in Figure 3. The total metabolite pools of all measured metabolites were reduced significantly (*p* = 0.029) with the exception of glutamine (*p* = 0.057), which was unchanged (Figure 3A). Incubating slices with 100 μmol/L AABA resulted in a significant decrease in incorporation of total 13C label into Glu C2, lactate C3 and Ala C3 (all *p* = 0.029), with no significant change in Glu C4, GABA C2, Gln C4 and Asp C2 and C3, and citrate C2 (Figure 3B). The decrease in net fluxes into Glu C2, lactate C3 and Ala C3 were mostly contributed by fluxes from [1-^13^C]d-glucose as label incorporation from [1,2-^13^C]acetate into these isotopomers was not significantly different to control, although a significant increase in label incorporation from [1,2-^13^C]acetate into Glu C4,5 (*p* = 0.029) was observed for the samples treated with AABA (Figure 3C,D).

### 3.4. Inhibition of Glutamine Transport with 10 mm GPNA under Depolarizing Conditions

**Figure 4:**
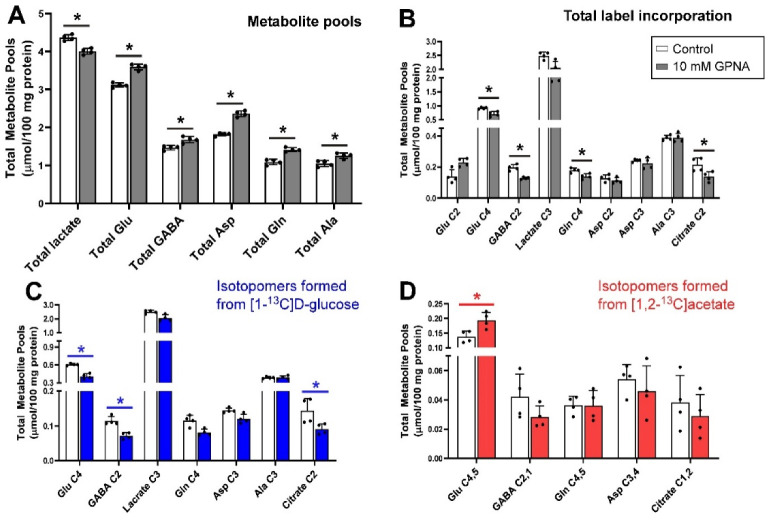
Distribution of ^13^C label following incubation of cerebellar tissue slices with 5 mmol/L [1-^13^C]d-glucose and 0.5 mmol/L [1,2-^13^C]acetate (control) and 10 mmol/L GPNA. (**A**), Total metabolite pools; (**B**), Total net fluxes of ^13^C; (**C**), Net fluxes derived from [1-^13^C]d-glucose; (**D**), Net fluxes derived from [1,2-^13^C]acetate. Bars show mean and error bars show standard deviations, N = 4. * *p* < 0.05 as shown with statistical significance (Mann–Whitney U test) between groups indicated by the line below the asterisk.

The addition of 10 mm GPNA (glutamine analogue) to slices resulted in significant increases in the total pool sizes of Glu, GABA, Asp, Gln and Ala (all *p* = 0.029) but a decrease in the pool of lactate (*p* = 0.029) (Figure 4A). In contrast, the net flux of ^13^C incorporated into Glu C4, GABA C2, Gln C4 and citrate C2 (all *p* = 0.029) isotopomers was significantly decreased by the administration of GPNA while there was no significant change in the total amount of ^13^C flux into Glu C2 (Figure 4B). Inspection of net fluxes from each of the labelled substrates showed that the decrease in flux into Glu C4, GABA C2 and citrate C2 was mostly contributed by the label from [1-^13^C]d-glucose (Figure 4C) as, except for the significant increase in net ^13^C flux into Glu C4,5, no significant change was measured in total label incorporation from [1,2-^13^C]acetate (Figure 4D).

### 3.5. Inhibition of Glutamine Transport with 10 mm cLeu under Depolarizing Conditions

**Figure 5:**
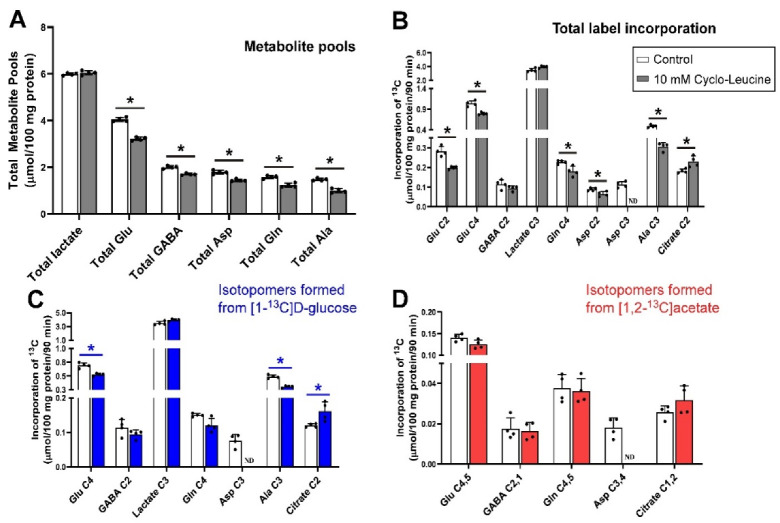
Distribution of ^13^C label following incubation of cerebellar tissue slices with 5 mmol/L [1-^13^C]d-glucose and 0.5 mmol/L [1,2-^13^C]acetate (control) and 10 mmol/L cLeu. (**A**), Total metabolite pools; (**B**), Total net fluxes of ^13^C; (**C**), Net fluxes derived from [1-^13^C]d-glucose; (**D**), Net fluxes derived from [1,2-^13^C]acetate. Bars show mean and error bars show standard deviations, N = 4. * *p* < 0.05 as shown with statistical significance (Mann–Whitney U test) between groups indicated by the line below the asterisk. ND = Not detected due to overlap of the resonance with that of c-Leu.

Administration of cLeu (10 mmol/L), an inhibitor of LAT1 and 2, to cerebellar slices significantly decreased the total pool sizes of Glu, GABA, Asp, Gln and Ala (Figure 5A). cLeu also decreased the net flux into Glu C2 and C4, Gln C4, Asp C2 and Ala C3 (all *p* = 0.029; Figure 5B) while there was a significant increase of net ^13^C flux into citrate C2 (*p* = 0.029). No significant change was observed in the total amount of ^13^C flux into lactate C3 or GABA C2 between control and cLeu treated slices. The resonance of Asp C3 (δ = 37.4 ppm) was obscured by overlap with the γCH2 resonance of c-Leu ((δ = 37.4 ppm) so no value is reported for this moiety.

Inspection of net fluxes from each of the labelled substrates indicated that the increase in flux into citrate C2 was mostly driven by flux from [1-^13^C]d-glucose as label incorporation from [1,2-^13^C]acetate into citrate C2,1 was not significantly different to control. cLeu also resulted in decreased incorporation of label from [1-^13^C]d-glucose into Glu C4 and Ala C3 (*p* = 0.029; Figure 5C) while again no significant changes were seen in the incorporation of label from [1,2-^13^C]acetate (Figure 5D).

### 3.6. Inhibition of Glutamine Transport with 10 mm BCH under Depolarizing Conditions

**Figure 6:**
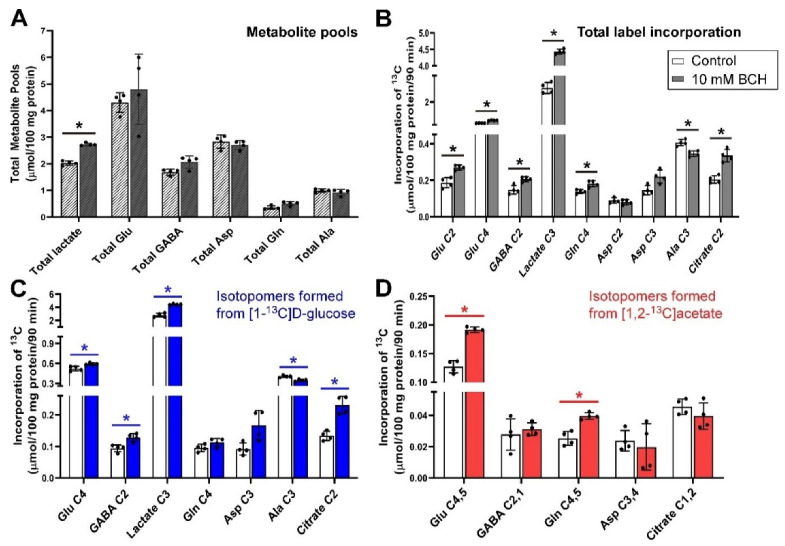
Distribution of ^13^C label following incubation of cerebellar tissue slices with 5 mmol/L [1-^13^C]d-glucose and 0.5 mmol/L [1,2-^13^C]acetate (control) and 10 mmol/L BCH. (**A**), Total metabolite pools; (**B**), Total net fluxes of ^13^C; (**C**), Net fluxes derived from [1-^13^C]d-glucose; (**D**), Net fluxes derived from [1,2-^13^C]acetate. Bars show mean and error bars show standard deviations, N = 4. * *p* < 0.05 as shown with statistical significance (Mann–Whitney U test) between groups indicated by the line below the asterisk. Hatched bars indicate that the pool sizes were derived from LCMS in this experiment rather than from ^1^H NMR due to co-resonant BCH resonances.

Incubation of cerebellar tissue slices with 10 mmol/L BCH had little impact upon total metabolite pools, apart from that of total lactate (*p* = 0.029) which was increased significantly (Figure 6A). BCH significantly increased net flux into Glu C2 and C4, GABA C2, lactate C3, Gln C4 and citrate C2, while the net flux into Ala C3 was significantly decreased (all *p* = 0.029, Figure 6B). Evaluation of net fluxes from the labelling substrates indicated that the rise in flux into Glu C4 and Gln C4 was contributed by both [1-^13^C]d-glucose and [1,2-^13^C]acetate. In contrast, the increase in net fluxes into GABA C2 and citrate C2 was mostly driven by flux from [1-^13^C]d-glucose as label incorporation from [1,2-^13^C]acetate into GABA C2,1 and citrate C1,2 was not statistically different compared to control (Figure 6D).

### 3.7. Visualization of Expression Data

**Figure 7:**
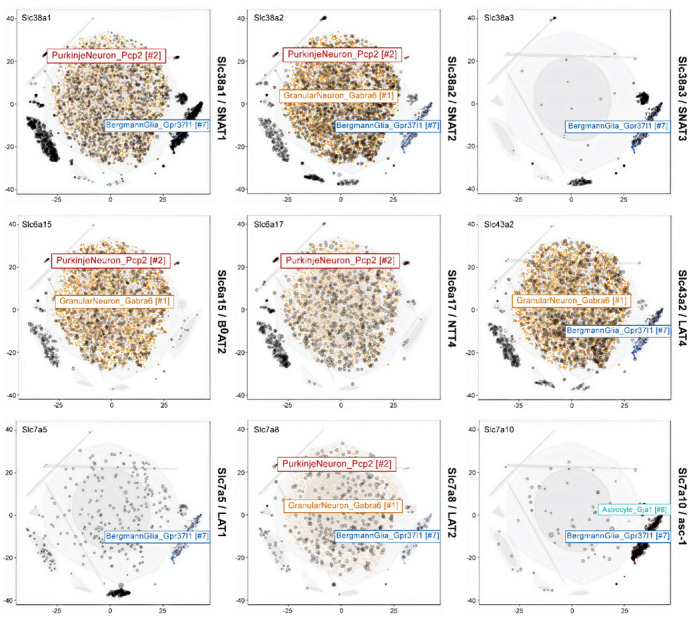
Visualization of different types of glutamine transporters in mouse cerebella using t-distributed stochastic neighbour embedding (tSNE). Each dot represents a cell, similar cells are grouped and shown in colours. The coloured dashed lines denote the major cell types.

To quantify cell type-specific gene expression patterns and to aid the interpretation of the experiments, we examined single cell transcriptomic databases [72] to analyse glutamine transporter expression in different cell types in the cerebellum (Figure 7, Table 2). We grouped these cell clusters into three categories: (1) Granular neurons, (2) Purkinje neurons, and (3) Bergmann glia. Inspection of the expression pattern showed that SNAT2 or Slc38a2 (*p* = 5.59e^−9^), LAT4 or Slc43a2 (*p* = 8.96e^−7^) and the branched-chain amino acid transporter B^0^AT2 or Slc6a15 (*p* = 2.94e^−21^) tended to cluster together within the granular neuron. However, SNAT2 or Slc38a2 was also found in Purkinje neurons and Bergmann glia. In agreement with earlier studies B^0^AT2/Slc6a15 and NTT4/Slc6a17 were found in both types of neurons but not in glial cells. The most Bergmann glia specific transporters were asc-1/Slc7A10 and SNAT3/Slc38a3. LAT1/Slc7a5 was also predominantly expressed in this cell type and absent in Purkinje neurons. LAT2 or Slc7a8 was the only glutamine transporter gene which was expressed within all the three cell types, i.e., granular neuron (*p* < 0.00), Purkinje neuron (*p* = 0.240) and Bergmann glia (*p* = 0.123).

## 4. Discussion

Histidine shares all its transporters with glutamine and as a result is a competitive inhibitor of system N (SNAT3) and A (SNAT1 and SNAT2) which are the major transport systems for recycling glutamine between astrocytes and neurons [30,60]. However, due to its structural similarity, it will also interfere with other transporters that accept glutamine. The decrease in pool sizes (Figure 1A) and net fluxes (Figure 1B) particularly in the labelling of Gln, C4 and GABA C2 show that these transporters play similar major roles in the cerebellum.

There were strong effects of histidine on both neuron and astrocyte metabolism. The impact on astrocyte metabolism can be seen in Figure 1D where there were large decreases in the incorporation of label from [1,2-^13^C]acetate into all isotopomers but particularly Gln C4,5. Glutamine from astrocytes has been shown to contribute to the synthesis of GABA in GABAergic neurons [90] and here, the amount of GABA labelled from [1,2-^13^C]acetate or from [1-^13^C]d-glucose was halved (Figure 1C,D).

Due to the ubiquitous presence of glutamate in all cells it is not possible to determine the exact contribution of label from glutamine to synthesis of glutamate in glutamatergic neurons, although by analogy with the above cycling of glutamine between astrocytes and GABAergic neurons and from studies using mathematical models [91] the contribution of glutamine to glutamate synthesis in these neurons could be relatively large. The increase in labelling of Glu C2, which is labelled on the second turn of the Krebs cycle, along with decreased net flux into Glu C4, which is mostly labelled on the first turn of the cycle, indicated the likely presence of a small Krebs cycle compartment with faster turnover along with the majority of Krebs cycling being comparatively slower. This can be inferred from the relative amount of increased Glu C2 labelling (second turn of the Krebs cycle) which is much smaller than the overall reduction in Glu C4 labelling (first turn of the cycle), from which it can be inferred that the increase in Glu C2 labelling does not arise simply from the overall rate of the Krebs cycle being faster.

The amount of total Asp in cerebellar slices was relatively small in contrast to that seen in the cortex under similar conditions [81,92] with the amount of labelled aspartate (C2 and C3) being negligible. This is in line with lower levels of aspartate reported in rat cerebellum vs. cerebrum [93]. Aspartate and alanine are usually in tight equilibria via the alanine and aspartate aminotransferases [94,95]. Here, the labelling of Ala C3 was significantly reduced by histidine (Figure 1C). Alanine cycling has been shown to be a key component of the glutamate/glutamine cycle, with alanine acting as a key molecule in the return of ammonia from the neuron to the astrocyte [8,9].

From the results of our study, it is seen that the decrease in pool sizes by MeAIB was the largest decrease seen with any inhibitor. Although histidine competes with glutamine, it is an able substrate, while MeAIB is transported very slowly thus causing stronger inhibition. In contrast to the cortex, inhibition of glutamine uptake by neurons with MeAIB in the cerebellum had a significant impact on metabolite pools indicating that SNAT1/SNAT2 play a key role in the cerebellum for maintaining metabolite levels. This is consistent with their role as amino acid loaders in other cell types [96]. In addition, the reduced incorporation of label into lactate could be explained by a reduced transfer of alanine between cells [9], producing less pyruvate to equilibrate with lactate, or just by the overall reduction in metabolism.

It is known that exogenous glutamine added to slices incubated with glucose and acetate greatly increases all metabolic pool sizes [2]. Loss of glutamine synthesis through inhibition of glutamine synthetase also greatly impacts pool sizes suggesting that the uptake of glutamine from the medium is key to maintaining neuronal metabolite pools. It has previously been shown that inhibition of glutamine uptake by MeAIB increases the concentration of glutamine in extracellular lysates [20] indicating that glutamine is released by glial cells but is unable to be taken up by neurons due to the inhibitor.

Although there was also a significant impact on the incorporation of label into glutamate and glutamine isotopomers, the impact on incorporation of ^13^C into GABA was relatively small. Indeed incorporation of label into GABA C2,1 from [1,2-^13^C]acetate was not significantly affected. It is known that GABAergic neurons can directly use [1,2-^13^C]acetate to make GABA C2,1 [81] which is what may be happening here. MeAIB also resulted in increased incorporation of label from [1,2-^13^C]acetate into citrate C2,1; this has also been shown to occur in cortex and hippocampus slices when glutamine synthetase is inhibited by methionine sulfoximine (MSO) [81,82].

AABA is an inhibitor of SNAT2 and LAT1 [56] but not SNAT1, which allows us to further dissect the impact of MeAIB, which inhibits both SNAT1 and SNAT2. The compound was originally developed as an anticancer drug and thought to inhibit ASCT2 [97]. From our study results, it was found that such blockade did not result in any significant change either in total metabolite pools or the net ^13^C flux into glutamine, but it significantly lowered the neurotransmitter (glutamate and GABA) pools. Again, the decreased flux of ^13^C from [1-^13^C]d-glucose into Glu C4 and increased flux of ^13^C from [1,2-^13^C]acetate into Glu C4,5 indicates that astrocytic glutamate to glutamine conversion is impaired and glutamine is taken up into the metabolically inactive compartments of the neurons which do not take part in neuronal glutamate synthesis.

Taken together, this suggests that a faster, small Krebs cycle, possibly in glial cells, is impacted by blocking SNAT2 and LAT1. There is a modest impact on pool sizes but this is less than that seen with MeAIB, suggesting that SNAT1 may be key in the uptake of amino acids for supplying total metabolic pools in neurons. We can also infer that SNAT2 and LAT1 play little role in the generation of GABA from glutamine in GABAergic neurons.

**Figure 8:**
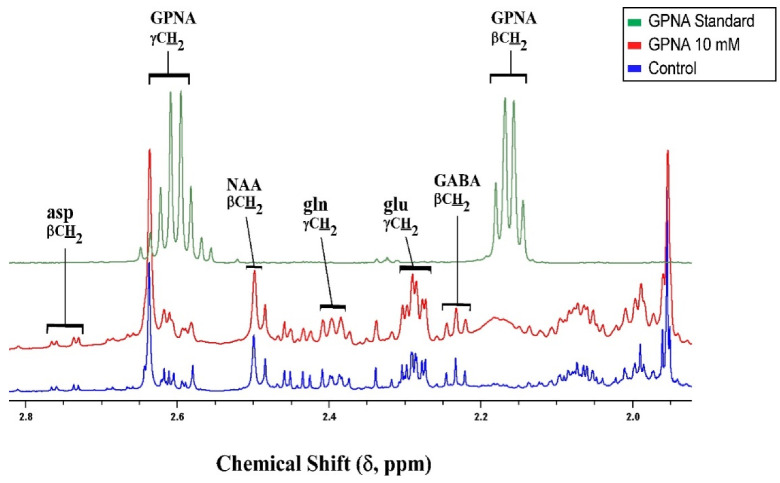
Example ^1^H 600.13 MHz NMR spectra extracted guinea pig cerebellum showing broadened resonances of GPNA. The broadened γ-glutamyl resonances from can be seen in the middle spectrum near δ = 2.2 and 2.6 ppm.

GPNA is a widely used inhibitor of ASCT2 [98] but kinetic studies have shown that GPNA is a low affinity, competitive inhibitor of system L transporters LAT1 and LAT2 [55] and that it also inhibits Na^+^-dependent neutral amino acid transporters of system A (SNAT1 and SNAT2) at higher concentrations [56]. GPNA is a substrate for γ-glutamyl transferase (GGT), releasing p-nitroaniline and glutamate after hydrolysis or in exchange with a range of amino acids. GGT activity in the cerebellum was reported to be around half of that in the cortex, although still sufficient to metabolise significant amounts of GPNA in the time frame of this experiment [99]. In the brain, γ-glutamyl transferase is an ectozyme [100] which would be expected to produce glutamate externally. Exogenous glutamate is known to activate metabolism in slices, resulting in increased pool sizes [101]. Inspection of the proton spectra from samples containing added GPNA showed a distinct broadening of the resonances of the γ-glutamyl moiety while all other resonances in the spectrum remained sharp and well shimmed (Figure 8), consistent with the γ-glutamyl moiety being conjugated with other ligands beside p-nitroaniline; indeed multiple candidates for γ-glu acceptors are present in the cerebellum [99].

Taken together, we can infer that the metabolic changes incurred by incubation of tissue slices with GPNA likely result more from byproducts of GPNA metabolism than from the activity of GPNA at glutamine transporters. If the latter were true, the metabolic profile produced by GPNA should be more similar to that generated by AABA as they block similar transporters. This aligns with the recent report of significant toxicity of *p*-nitroaniline in cancer cells over and above any effect of blockage of glutamine transport by GPNA [64]. The broad-spectrum inhibitor GPNA is therefore not recommended for the focused study of glutamine transport.

cLeu inhibits alanine exchange between astrocytes and neurons as well as that of glutamine. The decrease in aspartate pool size can be explained as a consequence of the impact on alanine cycling through the equilibration of aspartate and alanine aminotransferases which takes place in glial cells [95].

It should also be mentioned here that cLeu is an antagonist of the glycine binding site of the *N*-methyl-D-aspartate (NMDA) receptor [102] which might reasonably be expected to have a depressive effect on metabolism. Moreover, its specificity regarding amino acid transporters has not been tested extensively and the concentration used also may inhibit other transporters.

BCH is another glutamine transport inhibitor that acts on multiple transporters, i.e., it blocks neuronal B^0^AT2 [103] and both neuronal and astrocytic LAT1, LAT2 and LAT4 [2,12]. BCH is a known activator of glutamate dehydrogenase (E.C. 1.4.1.3; l-glutamate: NAD(P)^+^ oxidoreductase, deaminating) [104] which catalyses the conversion of glutamate to 2-oxoglutarate (α-ketoglutarate) and ammonia using NADP(H) as a cofactor. The enzyme is reversible but evidence suggests that in the brain it operates largely in the direction of production of ammonia, i.e., in the direction of glutamate oxidation [105]. There are two isoforms of GDH; GDH1, which is expressed widely including in the brain and mostly in mitochondria and GDH2, a human-specific isoform [106]. While BCH has been shown to increase the activity of this enzyme, it is not clear exactly what impact this might have on glutamate levels since GDH is but one (relatively highly regulated) enzyme that metabolises glutamate. Transaminases, such as the near-equilibrium enzymes aspartate aminotransferase and alanine aminotransferase also freely interconvert glutamate and 2-oxoglutarate which complicates attempts to interpret the system. It has been noted that the ratios of aspartate aminotransferase to glutamate dehydrogenase vary between different mitochondria in the brain [107] but the impact of this may be more important under conditions of substrate limitation which was not the case in our experiments.

Production of glutamate from glutamine is via phosphate-activated glutaminase, a mitochondrial enzyme found in neurons and product inhibited by glutamate [108]. One might expect, therefore, that increased activity of GDH might result in lower glutamate levels, reduced inhibition of phosphate-activated glutaminase and, by inference, lower levels of glutamine. Here, though, we saw little impact on the pool size of any metabolite apart from lactate (Figure 6A) and increased total net flux into GlnC4-containing isotopomers (Figure 6B); this latter increase was mostly due to increased incorporation of label from [1,2-^13^C]acetate. Indeed the relatively large increases in net flux into Glu C4,5, GABA C2,1 and Gln C4,5 indicates that flux from [1,2-^13^C]acetate, which is mostly metabolized in astrocytes, is strongly impacted by BCH although this does not extend to flux from [1,2-^13^C]acetate into citrate C2,1. Considered together with the increase in flux from [1-^13^C]d-glucose into Glu C4, GABA C2 and citrate C2 this would imply that the Krebs cycles in both neurons and glia were working harder in the presence of BCH. Taken together, the pattern of metabolism due to BCH implies blockage of glutamine labelled from [1-^13^C]d-glucose and glutamine labelled from [1,2-^13^C]acetate from leaving the astrocyte, which may cause increased metabolism of [1-^13^C]d-glucose, possibly in neurons, to compensate for this. The reduction in the cycling of glutamine is also supported by the increase in net flux into Asp C3 and citrate C2 indicating increases in the net flux of the astrocytic Krebs cycle.

In addition to blocking glutamine transfer via multiple transporters, BCH would also be blocking the transfer of alanine via LAT2. As alanine plays a key role in the return of ammonia from neurons to glia [8]. This may be expected to impact glutamine cycling as well as explain the significant decrease in the labelling of Ala C3 that was observed (Figure 6B,C). This decrease was also noted with cLeu (Figure 5B), which also blocks System L transporters but was not seen with AABA which is known to block LAT1 but has unknown efficacy at LAT2.

The presence of BCH significantly increased the metabolite pool of lactate and the net ^13^C flux into lactate C3. Given that the net flux is increased into Krebs cycle intermediates and metabolites and that the net flux into Ala C3 is decreased it would seem that the increased net flux into Lactate C3 is not likely to be due to decreased pyruvate clearance. However, given the known association between the activity of GDH and aspartate aminotransferase [107] with the resultant impact on the activity of the malate-aspartate shuttle [105] one explanation for the increase in lactate may be adjustment of the cellular redox (NAD^+^/NADH) ratio due to altered activity of the malate aspartate shuttle.

## 5. Conclusions

**Figure 9:**
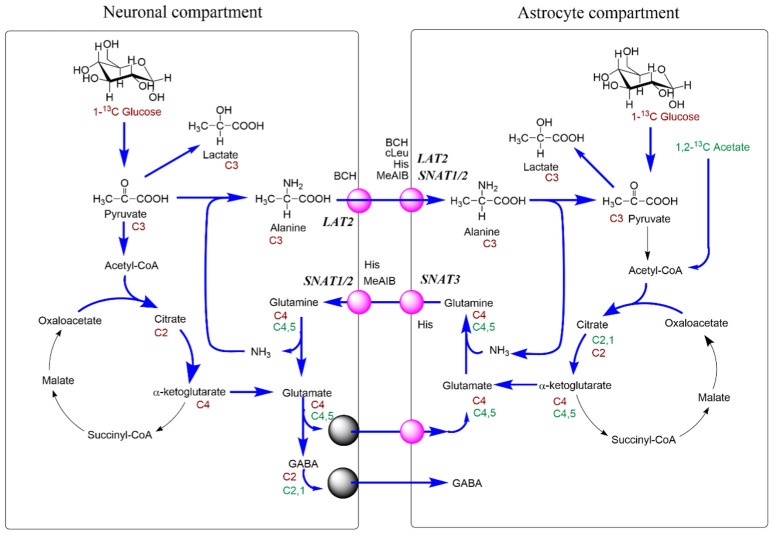
Overview of glutamine and alanine cycling in the cerebellum. Sites of transport inhibition by Histidine, MeAIB, cLeu, BCH are indicated.

Similar to results in the cortex [30], neurotransmitter recycling between neuronal and glial compartments was also observed in the cerebellum (Figure 9). In this study, we have used a wider variety of inhibitors but due to lack of specificity or non-specific toxicity results were not always straightforward to interpret. This was particularly true for the ASCT2 inhibitor GPNA, which we excluded from interpretation. BCH, cLeu and AABA have strong effects on the uptake of branched-chain amino acids, which are metabolised in neurons and glial cells. Increased TCA cycle activity in the presence of BCH may be explained by a reduced dilution of the label from BCAA remaining in the slices.

Overall, the effects of glutamine transporter inhibition were more pronounced in the cerebellum than in the cortex suggesting that glutamate/glutamine cycling is more extensive. MeAIB had a strong effect on fluxes into glutamate, while histidine was the only compound that affected fluxes into GABA. This suggests that SNAT1/2 may not be the dominant pathway for glutamine uptake into the synaptic compartment in Purkinje cells.

We could also confirm an important role of alanine in the return of amino groups from neurons to glial cells [9]. Regarding the expression of general amino acid transporters, we found little difference between GABAergic and glutamatergic cells; glial cells however have a transporter signature distinct from neurons.

The impact of glutamine cycling on brain function has been highlighted by the recent discovery of rare biallelic variants in *SLC38A3* [109], resulting in global developmental delay, microcephaly, epilepsy, absent speech and visual impairment. This underlines the importance of the astrocytic release of glutamine (Figure 9) which, it would appear, is unable to be compensated adequately by other transporters. Similarly, very localised ablation of glutamine synthetase using astrocyte-specific adeno-associated viral transfer has been shown to be sufficient to cause epileptic discharges [110] indicating that glutamine synthesis is needed for the regulation of excitatory activity.

An investigation that studied nerve terminals, isolated by transecting hippocampal Schaffer collaterals and cortical layer I axons, showed reduced quantal release of glutamate under conditions of stimulation. This was exacerbated by inhibition of astrocytic glutamine synthetase and restored by the addition of exogenous glutamine, implying that local nerve terminal glutamine uptake was required to maintain glutamate release [111]. The authors point out that glutamate release could be maintained under conditions of moderate stimulation but not indefinitely, pointing to a reservoir of glutamine or glutamate, or at least some local glutamate synthesis. Others have suggested that exogenously applied glutamine is only incorporated slowly into synaptically released glutamate and that neurons have sufficient local synthesis capacity to maintain glutamate synthesis without glutamate/glutamine cycling [112], despite some impact of inhibition of System A with MeAIB. Similarly, the amplitude and frequency of miniature IPSPs mediated by GABA are unaffected by exogenous glutamine, indicating that neurons can maintain GABA synthesis without relying on the uptake of glutamine. Recently, it has been suggested that the commonly used glutamine synthetase inhibitor, methionine sulfoximine (MSO) may also interfere with glutamate release at low concentrations [113] which, if validated, may require some revision of results obtained using MSO when measuring synaptic glutamate release.

When thinking about the glutamate/glutamine cycle, focusing on the synaptic compartment is possibly distracting as it is more likely that the pathway works more as a spiral than a cycle with multiple compartments involved. In the cerebellum, it would appear that the compartments served by SNAT1 play a major role in glutamine cycling between the cell types, based on the impact of MeAIB on both pool sizes and net metabolic fluxes at least in glutamatergic neurons (Figure 2). Histidine, which inhibits similar transporters to MeAIB but with less efficacy, also had impacts on pools and metabolic fluxes but also showed an impact on GABA labelling from [1,2-^13^C]acetate, unlike MeAIB (Figure 1D cf. Figure 2D). Based on kinetics (Table 1) and the degree of cerebellar expression (Figure 8; Table 2), we might expect that SNAT1 plays a major role in glutamine and alanine transport in the cerebellum along with SNAT3 and LAT2 (Figure 9). Our experimental data support this view.

## Figures and Tables

**Table 1:** Localization of different glutamine and alanine transporters in the cerebellum along with their properties.

SLC	Transporter Molecule	Transport System	Cerebellar Expression	Properties	Transport Mechanism	Affinity for Gln (K_M_)	Substrates Other than Gln	Inhibitors	References
SLC6A15	B^0^AT2	B^0^	Glutamatergic (pyramidal cells) and GABAergic neurons (Purkinje cells)	Electrogenic in nature; Na^+^-dependent and Cl^−^-independent; pH dependent	1:1 Na^+^/amino acid co-transport	5.3 ± 2.7 mm	Leu, Ile, Val, Prol, Met, Ala (K_M_ 670 ± 92 μM), and Phe. Gln is a poor substrate.	Aminoisobutyric acid, BCH, His, hydroxyproline, nipecotic acid, pipecolic acid, loratadine.	[21,39,47,48,49,50]
SLC6A17	NTT4	B^0^	Glutamatergic (synaptic vesicles) and GABAergic neurons (Purkinje cells)	Electrogenic in nature; Na^+^-dependent and Cl^−^-independent; pH dependent	1:1 Na^+^/amino acid co-transport	5.2 ± 1.5 mm	Leu, Ile, Val, Pro, Met, Gly, and Ala	Unknown	[18,22,49,51]
SLC7A5	LAT1	L	Astrocyte, neurons, and BBB	Na^+^- and pH independent	Obligatory exchanger (1:1 stoichiometry)	2.2 mm	Large neutral amino acids (LNAA), branched or aromatic AA	BCH, KYT-0353 (JPH203), and (Z)-4-chloro-N-(4-(trifluoromethoxy)phenyl)-5H-1,2,3-dithiazol-5-imine, GPNA, AABA	[2,44,52,53,54,55,56]
SLC7A8	LAT2	L	Astrocyte, neurons, and BBB	Na^+^- and pH independent	Obligatory exchanger (1:1 stoichiometry)	151–275 µM	Broad specificity small and large zwitterionic amino acidsAla K_M_ = 978 ± 143 μm	BCH, GPNA, cycloleucine	[2,9,44,52,55,57]
SLC38A1	SNAT1	A	Glutamatergic and GABAergic neurons	Electrogenic, Na^+^- and pH dependent	1:1 Na^+^/amino acid co-transport	230 µM	Gly, Ala (K_M_ = 0.3 ± 0.02 mm), Ser, Cys, Asn, His, Met	His, MeAIB, GPNA	[19,58,59,60,61,62,63,64,65]
SLC38A2	SNAT2	A	Glutamatergic and GABAergic neurons	Electrogenic, Na^+^- and pH dependent	1:1 Na^+^/amino acid co-transport	1.65 ± 0.27 mm	Gly, Pro, Ala (K_M_ = 529 ± 50 μM), Ser, Cys, Asn, His, Met	His, MeAIB, GPNA, AABA	[35,42,56,58,60,62,63,64,65,66]
SLC38A3	SNAT3	N	Astrocyte (Bergmann glia; granular cells; adjacent to glutamatergic, GABAergic, and glycinergic synapses)	Na^+^- and pH dependent; Bidirectional/reversible	1:1 Na^+^-AA cotransport; H^+^-antiport	2.4 mm	Asn, His	His, Ala Asn, Ser, Thr, Met, Leu, Trp, Val, Phe, Ile, Gly, Tyr, and Cys	[16,28,29,30,58,67]
SLC43A2	LAT4	L	Astrocyte and neurons	Na^+^ and Cl^−^ independent; pH-independent; shows two kinetic components	Uniport of AA	-	Neutral and branched chain AA (leucine, isoleucine, valine), methionine and phenylalanine	Leu, BCH, JPH203, Acivicin, 3-iodo-L-tyrosine, ESK242, ESK246	[46,68,69,70,71]

(S) symport, (A) antiport; K_m_ values are given for human isoforms. BCH (2-aminobicyclo-(2,2,1)-heptane-2-carboxylic acid); GPNA (L-γ-glutamyl-p-nitroanilide); MeAIB (α-(methylamino)-isobutyric acid); AABA (2-amino-4-bis(aryloxybenzyl)aminobutanoic acids).

**Table 2:** Relative expression of different transporter genes in cerebellar region.

Transporter	Name	Granule	Purkinje	Bergmann	Comment
Slc1a1	EAAT3	1.1	0	0	Neuronal Glu transporter
Slc1a2	GLT-1	1.79	1.61	5.56	
Slc1a3	GLAST	1.95	1.61	7.28	Glial Glu transporter
Slc1a4	ASCT1	0	0	2.3	
Slc1a5	ASCT2	0	0	0	
Slc1a6	EAAT4	0	4.84	0	
Slc1a7	EAAT5	nd	nd	nd	
Slc6a1	GAT1	0.69	1.1	4.17	GABA transporter
Slc6a5	GLYT2	nd	nd	nd	
Slc6a7	PROT	0	0.7	0	
Slc6a9	GLYT1	0.69	0.69	3.3	
Slc6a11	GAT3	0	0	3.2	
Slc6a12	BGT1	0	0	0	
Slc6a13	GAT2	0	0	0	
Slc6a14	ATB^0+^	nd	nd	nd	
Slc6a15	B^0^AT2	1.95	1.79	0	Neuronal BCAA transporter
Slc6a16	NTT5	nd	nd	nd	
Slc6a17	NTT4	1.61	2.77	0	Neuronal BCAA transporter
Slc6a18	B^0^AT3	nd	nd	nd	
Slc6a19	B^0^AT1	nd	nd	nd	
Slc6a20a	SIT	0	0	0	
Slc6a20b	SIT	nd	nd	nd	
Slc7a1	CAT1	0.69	1.1	0.69	Cationic AA transporter
Slc7a2	CAT2	0	0	1.79	Cationic AA transporter
Slc7a3	CAT3	0	0	0	
Slc7a4	CAT4	1.39	0.69	0	
Slc7a5	LAT1	0.69	0	1.39	Large neutral AA transporter
Slc7a6	yLAT2	0.69	0	0	
Slc7a7	yLAT1	0	0	0	
Slc7a8	LAT2	1.1	1.1	1.1	
Slc7a9	b^0+^AT	nd	nd	nd	
Slc7a10	asc1	0	0.69	3.9	Glial D-serine transporter
Slc7a11	xCT	0	0	0	Cystine/Glu exchanger
Slc7a12	asc2	nd	nd	nd	
Slc7a13	AGT1	nd	nd	nd	
Slc7a14		2.3	1.39	0	
Slc7a15		nd	nd	nd	
Slc16a10	TAT1	0	0	0	
Slc17a6	VGLUT2	0	0	0	Vesicular Glu transporter
Slc17a7	VGLUT1	3.2	1.1	0.69	Vesicular Glu transporter
Slc17a8	VGLUT3	nd	nd	nd	
Slc25a12	AGC1	2.56	2.64	1.1	Mito Asp/Glu carrier
Slc25a13	AGC2	0	0.69	0	Mito Asp/Glu carrier
Slc25a18	GC2	0	0	3.1	Mito Glu carrier
Slc25a22	GC1	2.56	2.2	0.69	Mito Glu carrier
Slc32a1	VIAAT	0	4.14	0	Vesicular GABA transporter
Slc36a1	PAT1	0.69	2.1	0.69	
Slc36a2	PAT2	0	0	0	
Slc36a3	PAT3	nd	nd	nd	
Slc36a4	PAT4	1.6	1.79	0.69	
Slc38a1	SNAT1	2.4	3.3	4.65	Small neutral AA transporter
Slc38a2	SNAT2	2.56	1.79	1.79	Small neutral AA transporter
Slc38a3	SNAT3	0	0.69	2.77	Gln transporter
Slc38a4	SNAT4	nd	nd	nd	
Slc38a5	SNAT5	0	0	0	Gln transporter
Slc38a6	SNAT6	0	0.69	0	
Slc38a7	SNAT7	0.69	0.69	1.1	Lysosomal AA transporter
Slc38a8	SNAT8	nd	nd	nd	
Slc38a9	SNAT9	1.1	1.4	0.69	Lysosomal AA transporter
Slc38a10	SNAT10	1.39	1.79	1.39	
Slc38a11	SNAT11	0	0	0	
Slc43a1	LAT3	nd	nd	nd	
Slc43a2	LAT4	2.2	0.69	1.95	Large neutral AA transporter

nd = not detected. Expression pattern is shown as different colours (green, high expression of individual transporters; yellow, co-expression; red, no expression). Values for differential overexpression are taken from dropviz.org [72] and are normalised mean log relative to the expression of all other genes in the cerebellum.

## Data Availability

Data from this work will be available shortly after publication via the UNSW data repository or via request to the corresponding author.
